# Multi-omics for unveiling potential antidiabetic markers from red, green and black mung beans using NIR-UPLC-MS/MS multiplex approach

**DOI:** 10.1038/s41598-025-03911-x

**Published:** 2025-06-01

**Authors:** Dina S. Ghallab, Reham S. Ibrahim, Doaa A. Ghareeb, Nihal M. El Newehy

**Affiliations:** 1 Department of Pharmacognosy, Faculty of Pharmacy, Alexandria University, Egypt, 21521 Alexandria Egypt; 2 Bio-screening and Preclinical Trial Lab, Biochemistry Department, Faculty of Science, Alexandria University, Alexandria, Egypt; 3 Center of Excellence for Drug Preclinical Studies (CE-DPS), Pharmaceutical and Fermentation Industry Development Center, City of Scientific Research and Technological Applications (SRTA-City), New Burg El-Arab City, Alexandria Egypt; 4 Research Projects unit, Pharos University, Alexandria, Egypt; 5 Department of Pharmacognosy, Faculty of Pharmacy, Damanhour University, Damanhour, Egypt

**Keywords:** Mung beans, Anti-diabetic, Fourier transform-near infrared (FT-NIR), Chemometrics, UPLC-MS/MS, Green chemistry, Biochemistry

## Abstract

Inspired by the nutritional and biological attributes of mung beans, the current work aims to monitor metabolome patterns of different mung species and their entanglements on antidiabetic potential using NIR-UPLC-MS/MS multiplex approach combined with chemometrics. In this regard, a total of 71 chromatographic peaks spanning sugars, amino acids, flavonoids, fatty acids and their lipid derivatives, and phytosterols were chemically profiled. Coincidently, OPLS-DA underscored an obvious discrimination among the green, red and black mung species suggesting their chemical discrepancies where eriodictyol-O-glucoside, caffeic acid, formononetin-O-glucoside, viniferal and genistin serve as focal discriminators of green mung beans while lysoPC 18:2, lanosterol, gallocatechin, tyramine, petunidin 3-O-glucoside, biochanin A, vigvexin A, vignatic acid B, lysoPC 16:0 and phaseollin were the determining metabolites of red ones. Successively, the differential markers enriched in black mung samples included 10-formyltetrahydrofolate, stearidonic acid, hydroxylinoleic acid, vignatic acid A, campestrol, arachidonic acid and PG (18:2/18:1). Experimentally speaking, all mung samples exerted noteworthy dose-dependent inhibitory potential towards α-amylase and α-glucosidase enzymes. OPLS coefficient plots highlighted gamma-aminobutyric acid (GABA), gallic acid and beta-sitosterol as possible efficacy metabolites harmoniously mediated antidiabetic potential. Equally important, NIR spectroscopic analysis coupled with PLS-R model quantitively predicted the bio-efficient markers from various mung bean samples with a significant level of experimental reliability. These findings pursue concept of nutritional therapy and provide a fresh perspective to probe into mung beans bioactive molecules which might serve as referenced templates for mitigating diabetes. However, future work should be explored to uncover muti-target mechanisms of mung beans-derived compounds and strengthen their relevance.

**Supplementary Information:**

The online version contains supplementary material available at 10.1038/s41598-025-03911-x.

## Introduction

Mung beans (*Vigna radiata* L., F. Fabaceae), also called as moong beans or green grammes, were traditionally consumed as a legume food globally for over 3500 years in African and Asian places^1^. Originally classified as *Phaseolus* aureus Roxb., a lot of species within the *Phaseolus* genus were later reclassified to the *Vigna* genus. The genus *Vigna* comprises nine crop species that are primarily cultivated for human consumption or as animal feed in regions ranging from tropical to temperate climates^2^. The most common species are green mung or green gram (*Vigna radiata* L.), black mung or black gram (*Vigna mungo* L.) and red mung or Azuki bean (*Vigna angularis* L.). Previous studies have indicated that bioactive compounds derived from legumes comprising proteins, carbohydrates, polyphenolics, dietary fibres, minerals, fatty acids, sterols and vitamins offer potential functional food in promoting good health, with their consumption increasing annually by 10%^3^. The World Health Organization (WHO) has also suggested that these bioactive compounds could improve healthcare and help combat numerous chronic degenerative diseases^4^. These compounds primarily function as antioxidants and have gained popularity recently due to their nutritional and therapeutic significance^5^. Mung beans have been shown to improve ailments such elevated blood pressure, cholesterol, and blood sugar, and to prevent cancer and skin pigmentation^6^. They also offer liver protection and boost the immune system. These health advantages are largely attributed to the presence and characteristics of the powerful mixture of active compounds found in mung beans^7^. The nutritional value of mung beans is highly regarded; by dry weight, they contain 20–25% protein. The main storage proteins in mung beans are globulin (60%) and albumin (25%). Consequently, alongside other cereals, mung beans have being consumed in greater quantities. Essential amino acids including leucine, isoleucine, phenylalanine, valine, tryptophan, arginine, methionine, and lysine are abundant in mung bean proteins. Therefore, mung beans are considered a significant source of dietary protein (240 g/kg)^8^. Mung beans are primarily composed of carbohydrates (55–65%, equivalent to 630 g/kg of dry weight), with starch being the main type. The remaining carbohydrates include raffinose, stachyose, and verbascose, which can cause flatulence^9^. Nevertheless, with sufficient soaking, fermentation, and germination, these oligosaccharides can be decreased and are readily soluble in water. Compared to other legumes, mung beans have less flatulence because their carbs are very easily digested. Additionally, mung beans and their sprouts are less calorically dense than other cereals, which makes them advantageous for people with diabetes or obesity. Additionally, tannins, hemagglutinin, trypsin inhibitors, phytic acid, and other antinutrients found in mung beans have a variety of biological functions that help the body get rid of toxins. Flavonoids (flavones, isoflavonoids, isoflavones, and anthocyanins), phenolic acids (gallic acid, ferulic acid, vanillic acid, caffeic acid, cinnamic acid, protocatechuic acid, p-hydroxybenzoic acid and shikimic acid), and other organic acids are among the numerous secondary metabolites that have been found to have health-promoting properties^10^. The most abundant flavonoids detected in the mung beans are Flavonols (kaempferol, quercetin, and myricetin) and flavones (luteolin, vitexin, isovitexin, and isovitexin-6″-O-α-l-glucoside). The two main flavonoids found in mung bean seeds are vitexin and isovitexin. Although they are mostly concentrated in the seed coats, these phenolic compounds are present in both the cotyledons and the seed coats. Furthermore, a number of variables, including cultivar, seed coat colour, meteorological and agronomic circumstances throughout growth, and extraction and analysis techniques, affect the amount and makeup of bioactive chemicals in mung beans^7^.

Diabetes Mellitus (DM) significantly impacts the wellbeing and quality of life of millions globally. While most DM patients have Type 2 Diabetes^11^. A key therapeutic strategy for managing Type 2 DM consists of preventing the conversion of dietary carbohydrates into simple sugars in order to lower postprandial blood glucose levels, thereby preventing their absorption^11^. The enzymes α-glucosidase and α-amylase, which hydrolyze carbohydrates, are the primary targets of this discipline. Mung beans has emerged as a promising agent in this context^8,12-15^, especially when used by the suitable processing methods as boiling and sprouting, while vitexin and isovitexin are the two phenolic compounds responsible for the high antidiabetic activity^16,17^. In this regard, mung bean starch is notable for its low glycemic index. Therefore, its application in the creation of such novel products can aid in reducing the risk of diabetes and managing obesity^18^. But, its standardization depending on metabolomics analysis is essential to ensure perfect chemical consistency and reliable efficacy^19^. It is worth noting that there is no previous comprehensive qualitative and quantitative metabolomics study on green, black and red mung bean species along with their antidiabetic efficacies. Therefore, this study aims to standardize mung bean samples collected from the three common species. To achieve this, an integrated strategy for enhanced quality control has been implemented. Fingerprint analysis of mung bean samples using Fourier Transform-Near-Infrared spectroscopy (FT-NIR) in addition to comprehensive chemical profiling using UPLC-QqQ-MS technique were established. By combining these two independent techniques with multivariate analysis, a thorough and precise discrimination, along with high-quality assessment, of mung samples collected from various species was achieved. Furthermore, a fingerprint-efficacy relationship analysis was established utilizing an Orthogonal Projection to Latent Structure (OPLS) multivariate model to identify bio-efficient antidiabetic biomarkers. Finally, for the rapid and accurate prediction of efficacy-related biomarkers a Partial Least Squares Regression (PLS-R) model was developed using FT-NIR data as independent variables.

## Results and discussion

### Metabolites assignment in differently coloured mung beans extracts

**Fig. 1:**
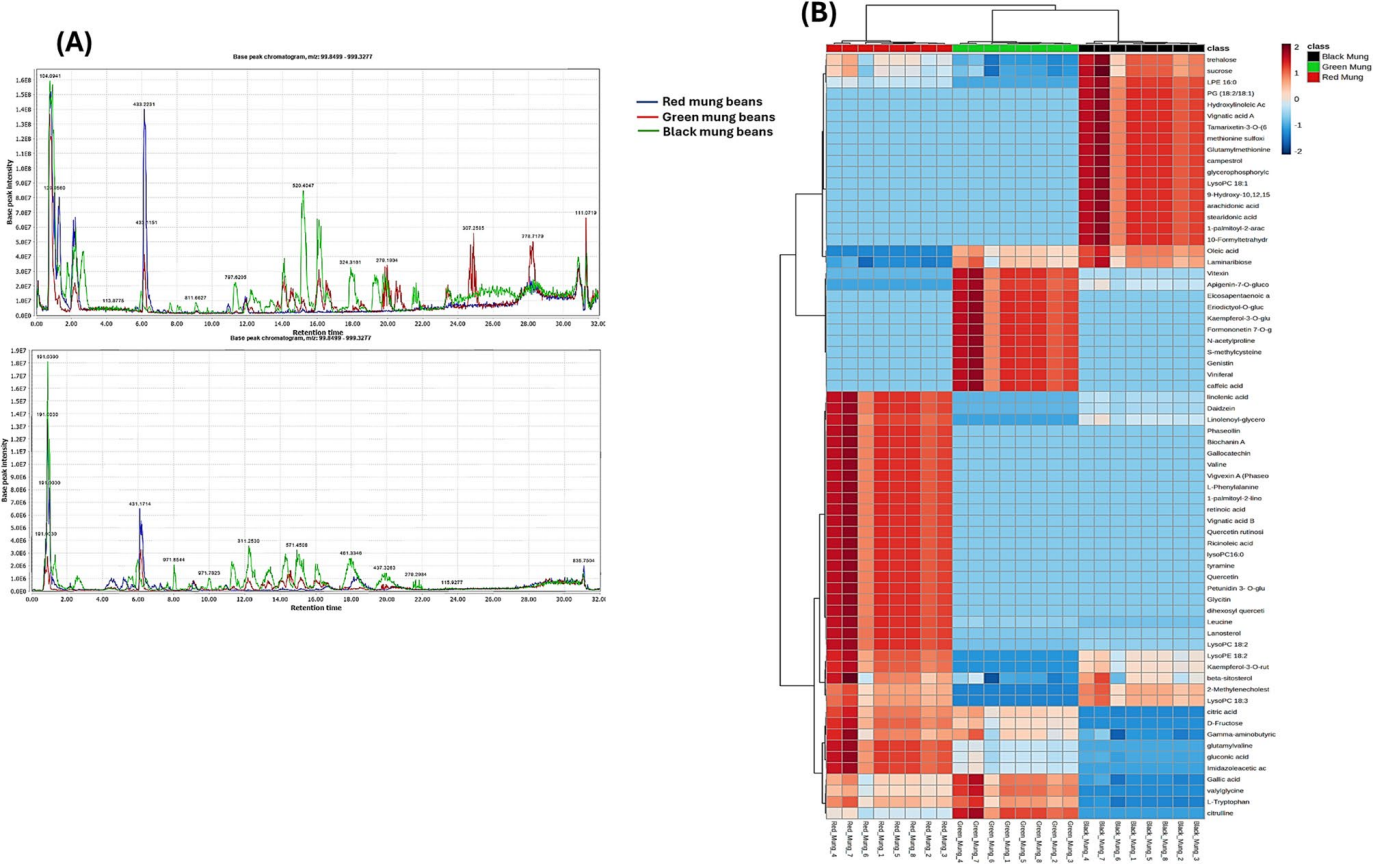
Respective base peak chromatograms (BPCs) retrieved from different mung species in both positive and negative ionization modes (**A**). Heat map of all identified compounds in various mung samples with the relative amount of metabolites from high to low represented by a color-coded scale grading from red to blue (**B**).

In the current investigation, UPLC-QqQ-MS/MS metabolomics approach was incorporated to objectively demonstrate compositional heterogeneity among differently coloured mung beans (red, green and black) samples in a holistic viewpoint. In this regard, a total of 71 chromatographic peaks spanning sugars, amino acids, dipeptides, flavonoids, anthocyanins, phenolic acids, fatty acids, phospholipids and phytosterols were chemically annotated relying on the respective retention time and unique fragments profiles in conjunction with reference standards and pertinent literature. Base peak chromatograms (BPCs) of the investigated samples in positive and negative polarity modes were depicted in Fig. 1A. In a similar vein, Table 1 briefly outlines the complete list of annotated compounds derived from the various mung samples, their corresponding mass data retention times, molecular formula, and chemical classes.

**Table 1:** List of annotated compounds extracted from differently colored mung beans species.

No.	Rt (min)	Compound name	Precursor ions	Chemical formulas	Mass fragments	Reference	Chemical class	Source
1	0.734572	Sucrose	M-H 341.3	C_12_H_22_O_11_	179, 161, 119, 113	(1)	Sugars	All samples
2	0.740356	Trehalose	M-H 341.T	C_12_H_22_O_11_	179, 161, 113	(2)	Sugars	All samples
3	0.754825	D-Fructose	M-H 179.18	C_6_H_12_O_6_	161, 149, 113,	(1)	Sugars	Red & green mung beans
4	0.754825	Laminaribiose	M-H 341.3	C_12_H_22_O_11_	179, 161, 149, 113	(3)	Sugars	All samples
5	0.772167	Glutamylmethionine; L-L-form, S-Oxide	M + H 295.4	C_10_H_18_N_2_O_6_S	231, 150, 147,	(4)	Amino acids	Black mung beans
6	0.777961	Gamma-aminobutyric acid (GABA)*	M + H 104.2	C_4_H_9_NO_2_	87, 60	(5)	Amino acids	All samples
7	0.804	Gluconic acid	M-H 195.2	C_6_H_12_O_7_	151, 133	(6)	sugars	All samples
9	0.8069	valylglycine	M + H 175.3	C_7_H_14_N_2_O_3_	101, 75	(7)	Dipeptides	Red & green mung beans
10	0.858983	Glycerophosphorylcholine (GPC)	M + H 258.3	C_8_H_20_NO_6_P	240, 184, 104, 86	(8)	Glycerophosphorylcholine	Black mung beans
11	0.858983	Methionine sulfoxide	M + H 166.2	C_5_H_11_NO_3_S	138, 120, 56	(9)	Amino acids	Black mung beans
12	0.858983	Glutamyl valine	M + H 247.3	C_10_H_18_N_2_O_5_	229, 186, 146, 128	(10)	Amino acids	Red & green mung beans
13	0.876333	Methyl cysteine	M + H 136.3	C_4_H_9_NO_2_S	119, 104, 90, 74	(7)	Amino acids	Green mung beans
14	0.893683	Valine	M + H 118.2	C_5_H_11_NO_2_	72	(7)	Amino acids	Red mung beans
15	0.902383	Citric acid	M-H 191.2	C_6_H_8_O_7_	173, 147, 103	(11)	Organic acids	All samples
16	0.902383	Imidazole acetic acid	M-H 125.3	C_5_H_6_N_2_O_2_	79, 41	(12)	Organic acids	Red & green mung beans
17	1.032567	Citrulline	M + H 176.2	C_6_H_13_N_3_O_3_	159, 132, 116, 70,	(13)	Amino acids	Red & green mung beans
18	1.310317	N-acetyl proline	M + H 158.3	C_7_H_11_NO_3_	140, 115, 70	(14)	Amino acids	Red mung beans
19	1.318983	Gallocatechin	M-H 305.3	C_15_H_14_O_7_	261, 179, 125	(15)	Tannins	Red mung beans
20	1.605417	Tyramine	M + H 138.2	C_8_H_11_NO	121, 107, 91, 65	(16)	Amino acids	Red mung beans
21	2.189855	L-Tryptophan	M + H 205.2	C_11_H1_2_N_2_O_2_	188, 161, 146, 130, 117	(7)	Amino acids	All samples
22	2.207211	Gallic acid*	M + H 171.2	C_7_H_6_O_5_	153, 143, 127,	(15)	Phenolic acids	All samples
23	2.5428	L-Phenylalanine	M + H 166.3	C_9_H_11_NO_2_	166, 149, 120	(7)	Amino acids	Red mung beans
24	5.17275	Eriodictyol-O-glucoside	M-H 449.3	C_21_H_22_O_11_	287, 243, 151, 135	(17)	Flavonoids	Green mung beans
25	5.433133	Caffeic acid	M-H 179.2	C_9_H_8_O_4_	135–143	(18)	Phenolic acids	Green mung beans
26	5.6588	Kaempferol-3-O-glucoside (Astragalin)	M-H 447.4	C_21_H_20_O_11_	284, 285, 255, 447	(6)	Flavonoids	Green mung beans
27	5.67615	Dihexosyl quercetin	M-H 625.4	C_27_H_30_O_17_	463, 301, 505, 179, 151	(19)	Flavonoids	Red mung beans
28	5.936533	Petunidin 3- O-glucoside	M-H 478.4	C_22_H_23_O_12_^+^	477, 315, 301	(20)	Anthocyanins	Red mung beans
29	5.936533	Glycitin	M-H 445.3	C_22_H_22_O_10_	283, 268, 224, 133	(21)	Flavonoids	Red mung beans
30	5.945217	Biochanin A	M + H 285.3	C_16_H_12_O_5_	271, 227, 152, 123	(22)	Flavonoids	Red mung beans
31	6.092767	Rutin (Quercetin − 3O-rutinoside)	M-H 609.4	C_27_H_30_O_16_	301, 272, 255, 151	(23)	Flavonoids	Red mung beans
32	5.962584	Quercetin	M + H 303.2	C_15_H_10_O_7_	273, 257, 151, 135	(18)	Flavonoids	Red mung beans
33	6.118808	Apigenin-O-glucoside (Apigetrin)	M-H 431.3	C_21_H_20_O_10_	269, 241, 151, 117, 107	(24)	Flavonoids	black & green mung beans
34	6.136158	Vitexin (Apigenin -C-glucoside)	M + H 433.3	C_21_H_20_O_10_	313, 283, 271, 153, 147, 119	(23)	Flavonoids	Black & green mung beans
35	6.1709	Formononetin -O-glucoside (Ononin)	M + H 431.3	C_22_H_22_O_9_	269, 254, 137, 153	(25)	Flavonoids	Green mung beans
37	6.787133	Viniferal	M-H 473.5	C_35_H_26_O_8_	527, 453, 395	(26)	hydroxystilbenoid	Green mung beans
38	7.255833	Genistin	M-H 431.3	C_21_H_20_O_10_	268, 269, 431	(27)	Flavonoids	Black & green mung beans
39	12.20322	Daidzein	M + H 255.3	C_15_H_10_O_4_	237, 153, 137	(21)	Flavonoids	black & red mung beans
40	13.0191	10-Formyltetrahydrofolate	M-H 472.4	C_20_H_23_N_7_O_7_	297, 178	(28)	Organo-heterocyclic compounds	Black mung beans
41	13.31419	Kaempferol-3-O-rutinoside (Nictoflorin)	M-H 593.3	C_27_H_30_O_15_	285, 257, 151,447,199	(26)	Flavonoids	black & red mung beans
42	13.32288	Vigvexin A (Phaseol)	M + H 337.4	C_20_H_16_O_5_	293, 249	(29)	Coumestans	black & red mung beans
43	13.81762	Tamarixetin-O-(6’’-malonyl) glucoside	M-H 563.3	C_25_H_24_O_15_	300, 255, 163, 151	(22)	Flavonoids	Black mung beans
44	14.01725	Vignatic acid B	M + H 520.3	C_27_H_41_N_3_O_7_	476, 364, 112	(30)	Cyclic peptides	Red mung beans
45	14.12141	LysoPC 18:3	M + H 518.3	C_26_H_48_NO_7_P	239, 257, 279, 235, 59	(31)	Lysophosphatidylcholines	black & red mung beans
46	14.50332	Stearidonic acid	M + H 277.4	C_18_H_28_O_2_	255, 233, 162	(32)	Fatty acids	Black mung beans
47	14.5988	9-Hydroxy-10,12,15-octadecatrienoic acid	M-H 293.3	C_18_H_30_O_3_	275, 235, 171	(33)	Fatty acids	Black mung beans
48	14.84182	LysoPE 16:0	M + H 454.3	C_21_H_44_NO_7_P	257, 213, 215, 197	(32)	lysophosphatidylethanolamines	black & red mung beans
49	15.04143	LysoPE 18:2	M-H 476.3	C_23_H_44_NO_7_P	279.3	(34)	lysophosphatidylethanolamine	black & red mung beans
50	15.13691	LysoPC 18:2	M + H 520.4	C_26_H_50_NO_7_P	281, 241, 237	(31)	lysophosphatidylcholines	black & red mung beans
51	15.66637	linolenic acid	M + H 279.3	C_18_H_30_O_2_	237, 113, 69	(31)	Fatty acids	black & red mung beans
52	15.67507	Hydroxy linoleic Acid	M-H 295.3	C_18_H_32_O_3_	279, 237, 59	(31)	Fatty acids	Black mung beans
53	16.00487	lysoPC16:0	M-H 494.3	C_24_H_50_NO_7_P	239, 257, 212	(31)	lysophosphatidylethanolamines	Red mung beans
55	16.4215	beta-sitosterol*	M + H 415.6	C_29_H_50_O	397, 273, 161, 107	(31)	Sterols	All samples
56	16.51697	Phaseolin	M + H 323.4	C_20_H_18_O_4_	305, 277, 195	(35)	Lipids	Red mung beans
57	17.654	Vignatic acid A	M-H 552.5	C_30_H_39_N_3_O_7_	508, 418, 392	(30)	Cyclic peptide	black mung beans
58	18.01854	Lanosterol	M-H 425.6	C_30_H_50_O	123, 109, 95	(31)	Sterols	black & red mung beans
59	18.06192	Linolenoyl-glycerol	M-H 351.3	C_21_H_36_O_4_	277, 233, 59	(31)	Lipids	black & red mung beans
60	18.47857	LysoPC 18:1	M + H 522.5	C_26_H_52_NO_7_P	283, 239, 124, 59	(31)	Lysophosphatidylcholines	black mung beans
61	19.25972	Retinoic acid	M + H 301.4	C_20_H_28_O_2_	257, 241, 121	(31)	Vitamins	Red mung beans
62	19.88465	Campestrol	M + H 401.7	C_28_H_48_O	383, 268, 161, 147	(32)	Sterols	black mung beans
63	19.902	Ricinoleic acid	M + H 299.4	C_18_H_34_O_3_	283, 239, 124, 59	(31)	Fatty acids	Red mung beans
64	20.66582	Arachidonic acid	M + H 305.4	C_20_H_32_O_2_	261, 207	(14)	Fatty acids	black mung beans
65	20.96958	Eicosapentaenoic acid	M-H 301.1	C_20_H_30_O_2_	259, 108	(31)	Fatty acids	Green mung beans
66	23.39989	2-Methylenecholestan-3-ol	M + H 401.4	C_28_H_48_O	383, 369, 189	(36)	Sterols	black & red mung beans
67	23.8599	1-palmitoyl-2-linoleoyl-GPC (16:0/18:2) (lecithin)	M-H 757.2	C_42_H_80_NO_8_P	501, 519, 478, 496, 279, 255	(37)	Glycerophosphorylcholine	Red mung beans
68	23.91	Oleic acid	M-H 281.3	C_18_H_34_O_2_	237-124-59	(32)	Fatty acids	black & green mung beans
69	25.76943	PG (18:2/18:1)	M + H 774.2	C_42_H_77_O_11_P	494, 492, 519, 281	(38)	phosphatidylglycerols	black mung beans
70	26.7589	1-palmitoyl-2-arachidonoyl-GPC (16:0/20:4)	M-H 781.3	C_44_H_80_NO_8_P	526, 544, 479, 497,303.255	(39)	Glycerophosphorylcholine	black mung beans
71	28.11292	PG (18:0/18:0)	M-H 778.4	C_42_H_83_O_10_P	495, 513, 490, 471, 283, 239	(38)	phosphatidylglycerol	black mung beans

*Identified by comparison with authentic references.

Complementarily, the relative average content of main chemical classes monitored in different mung species was depicted in stacked bar chart (Fig. S1**).**

In a broader sense, the following subsections provide the scheme utilized to annotate the key chemical classes in the different mung samples. Crucially, precision and reproducibility of the analytical system were assessed through QC samples.

### Sugars

Five peaks were detected under sugars category including 3 disaccharides (**1**,** 2 & 4**), one monosaccharide (**3**) and one sugar acid (**7**).

Trehalose was the suggested for peak (**2**) recorded at m/z [M – H]^−^ 341.21. Upon MS/MS analysis, two prominent daughter ions at m/z 179 and 161 consistent with the glycosidic cleavage at C1 and C3 forming two hexose units were assigned which sequentially fragmented through water loss.

Peak (**4**) was annotated as laminaribiose at m/z [M – H]^−^ 341 undergoes characteristic glycosidic bond cleavages, cross-ring fragmentations (Characteristic of Disaccharides), and neutral losses, especially of water and sugar-related fragments.

Peak (**7**) revealed an intense deprotonated precursor ion [M − H]^−^ at m/z 195.3 combined with fragment signals at m/z 151 and 133 attributable to the consecutive elimination of CO_2_ and H_2_O moieties.

### Organic acids

Three peaks (**6**,** 15 & 16**) assigned as organic acids were recorded in the current chromatographic runs.

Compound (**6**) exhibited a protonated signal at m/z 104.2 and daughter ions at m/z 87 and 60 matching with continuous losses of NH_3_ and CO_2_ moieties. Correspondingly, peak (**6**) was annotated as γ-amino butyric acid further confirmed by reference standard data.

Compound (**15**) forms a deprotonated ion at m/z 191.2 ([M–H]⁻) and fragments via decarboxylation (-CO₂, m/z 147) and dehydration (-H₂O, m/z 173). Further CO₂ loss produces m/z 103, confirming a characteristic citric acid fragmentation pattern.

Regarding compound **16** of m/z 125.1, it was characterized as imidazole acetic acid. The characterization was farther confirmed by the MS^2^ data, which unveiled a distinctive product ion at m/z 79 upon decarboxylation along with a minor ion at m/z 41 assigned to C_2_H_3_N upon on imidazole ring fission. This data aligned with an earlier record^20^.

### Amino acids, dipeptides and cyclic peptides

Amino acids (AAs) are a basic class of primary metabolite mostly found in mung beans and contribute to their unique nutritional^21^.

Under the current investigation, nine amino acids (**5**,** 11**,** 13**,** 14**,** 17**,** 18**,** 20**,** 21 & 23**) as well as two dipeptides (**9 & 12**) were detected during mass spectra examination.

From a practical standpoint, a prominent peak (**11**) found at m/z 166.3 [M + H]^+^ was tentatively annotated as methionine sulfoxide further confirmed by relevant data^22^. During its MS^2^ analysis, two informative ions at m/z 138 and 120 coherent with consecutive loss of CO and NH_3_ were detected. Further, a distinctive daughter ion of m/z 56 progressively ascribable to an additional loss of CH_3_SO was recorded.

Alongside, the acquired m/z values demonstrated the occurrence of valine and citrulline assigned in signals (**14 & 17**), respectively and had comparable fragmentation paths. essentially manifested with series of neutral elimination of H_2_O, CO succeeded by a major product ion consistent with NH_3_ loss.

Comparably, an acylated amino acid (**18**) displaying a quasi-molecular ion [M + H]^+^ at m/z 158.3 was putatively annotated as acetyl proline. During its MS^2^ analysis, it initially formed a promising ion coherent with distinguishing loss of acetyl unit (-43 Da) expelling the respective amino acid at m/z 115 further fragmented upon a collateral loss of NH_3_ + CO yielding a sharp product signal at m/z 70. Considering the above mass data and previous literature data ^23,24^, we can conclude that component **18** might be acetyl proline.

Compound (**21**) forms a quasi-molecular ion [M + H]⁺ at m/z 205.2, displaying a characteristic L-Tryptophan fragmentation pattern. It undergoes deamination (-NH₃, m/z 188) and decarboxylation (-CO₂, m/z 161). Further indole ring fragmentation produces m/z 146, 130, 117, and 103, confirming its structural identity.

Regarding dipeptides, a compound (**9**) recorded at m/z 175.3 [M + H]^+^ was identified as valyl glycine. Its CID-MS/MS initially gave rise to a fragment ion of 101 Da (valine moiety) through the protonated amide bond joint cleavage in the respective dipeptide revealing a prominent ion at m/z 75 furtherly dissociated through an accompanying elimination of NH_3_ + CO.

Furthermore, two cyclic peptides with representative peaks; **44 & 57** were clearly detected and respectively annotated as vignatic acid B and vignatic acid A representing chemotaxonomic markers of mung beans.

Peak **44** exhibited an intense molecular ion at m/z 520.2 for [M + H]^+^ along with a prime product ion of m/z 476 forming from loss of CO_2_. Also, amide bond cleavage expelled N-terminal and C-terminal fragments at m/z 364 and 112, respectively. Considering the mass data stated above, compound **44** could be characterized as vignatic acid B.

### Phenolic acids, flavonoids, anthocyanins and coumestans

Phenolic acids and flavonoids constitute vast groups of metabolites significantly existing in mung beans^25^.

In current chromatographic runs, only two phenolic acids exemplified in signals (**22 & 25**) were chemically annotated.

Peak (**22**) displayed (m/z 171 [M + H]⁺) fragments through neutral losses of H₂O (-18 Da, m/z 153), CO (-28 Da, m/z 143), and CO₂ (-44 Da, m/z 127). Further breakdown of the aromatic system produces smaller ions at m/z 109, 97, and 81, characteristic of phenol-derived structures. This pattern helps in the annotation of gallic acid.

The suggested candidate for signal (**25**) was caffeic acid owing to the molecular ion at m/z 179.2 and the typical ions of m/z 135 and 143 corresponding with CO_2_ and two H_2_O units loss.

In addition, a total of 16 flavonoid peaks pertaining the four subclasses primarily flavonols, flavone, flavanones, and flavan-3-ols along with some glycosylated forms were found constituting one of the main groups of metabolites found in the studied mung species. As well, three isoflavonoids were noted in the acquired MS spectra.

For eriodictyol-O-glucoside (**24**), the deprotonated ion [M − H]^−^ at m/z 449.3 along with a dominating aglycone (base peak) at m/z 287 [M - H − 162] ^−^ resulted upon hexose moiety loss were monitored. During MS^2^, a daughter ion at m/z 243 was formed by loss of CO_2_ (− 44 Da) from the C ring. Further, retrocyclisation pathway for C-ring expelled two major signals of m/z 135 [^1,2^A]^−^ and 151 [^1,3^A]^−^.

The metabolite at signal **(29**) revealed a dominant precursor ion [M − H]^−^ at m/z 445.2. Its MS^2^ profile dissected the occurrence of a major ion at m/z 283 [M–H–162]^−^ coming from neutral loss of glycosyl residue. Glycitein was affirmed as aglycone by the distinctive ions at m/z 268 and 224 gained through consecutive elimination of methyl radical and CO_2_. Also, a typical RDA ion [^1,3^B]^−^ of m/z 133 was found suggesting the occurrence of an additional hydroxyl group on ring-A.

In positive polarity mode, the ion at m/z 285.25 corresponded to biochanin A (Peak **30**). During MS^2^ experiment with higher collision energy, main product ions at m/z 271, 227, 152 and 123 respectively formed as a result of methyl radical loss, CO_2_ and retro-Diels-Alder (RDA) fission. The fragmentation profile recorded for biochanin A were in good alignment with earlier literature data^25^.

Furthermore, compound (**34**) (m/z 433 [M + H]⁺) undergoes characteristic C-glycoside fragmentation. It first undergoes cross-ring cleavage of glucose, producing m/z 343 (-90 Da) and m/z 313 (-120 Da). The loss of the glucose moiety (-162 Da) yields m/z 271, corresponding to the apigenin aglycone. Further fragmentation of the flavone core generates m/z 153, 147, and 119, confirming the structural identity of vitexin (Apigenin -C-glucoside). This pattern is essential for distinguishing C-glycosides from O-glycosides in natural product analysis.The fragment ion spectrum of the deprotonated compound **43** of m/z 563.15 [M–H]^−^ revealed a base peak with m/z 477 formed upon cleavage of malonyl moiety (– 86 Da) along with an ion at m/z 315 after hexose moiety (–162 Da). Tamarixetin was assured as aglycone part by the distinguishing fragment ions of m/z 300, 255, 163 and 151 formed at high collisional energy accounting for neutral elimination of CH_3_ radical and CO_2_ along with RDA fragments. Building on the analyses above and the pertinent literature data ^24,25^, compound **43** might be identified as tamarixetin-3-O-malonylglucoside.

One anthocyanidin with a respective peak; **28** was clearly noted under the applied ESI conditions.

Peak **(28**) could be assigned as petunidin 3-O-glucosideon based on its precursor ion at m/z 477.35 for [M − 2 H]^-^ and a principal aglycone signal at m/z 315 formed after the loss of neutral hexose unit. This aglycone was confirmed as petunidin by its MS^2^ analysis manifested by characteristic daughter ions at m/z 301, 273, 203 and 137 further confirmed from previous records^26^.

Further, one coumestan peak (**42**) was recorded and annotated as vigvexin A (Phaseol) based on its protonated molecular ion [M + H]^+^ at m/z 337.3 and dominating mass fragments at m/z 293 and 249 sequentially matching with losses of CO_2_ and isopropyl (43 Da) moieties further confirmed from previous literature data^27^.

### Fatty acids

Under the current analysis, fatty acids especially unsaturated forms sharply predominate the secondary metabolites in the investigated mung species (Table 1).

Peak **46** revealed mass characteristics consistent with stearidonic acid displaying a parent ion [M + H]^+^ at m/z 277.3 and major product ions at m/z 255 and 233 arising from water and CO_2_ moieties loss. As well, fissure of double bond C6-C7 of parent ion generated an extra product signal at m/z 162. MS^2^ profile outlined above together with the previous data^28^ affirmed peak **46** identity.

Peak **59** representing fatty acid glyceride was clearly detected under the current analysis and tentatively assigned as linolenoyl-glycerol. This signal gave a parent ion [M-H]^−^ of m/z 353.3 as well as daughter ions at m/z 277, 233 and 59 matching with linolenic acid formation and its distinctive fragments after favourable elimination of glycerol head group and further affirmed by earlier reports ^2428^.

The compound at signal **68** was matched to oleic acid with a molecular ion [M − H]^−^ at m/z 281.3 and a dominating fragment at m/z 237 after loss of CO_2_. Tandem MS fragments at m/z 124 (C_9_H_16_) [M − H – C_9_H_16_O_2_]^−^ and m/z 59 (C_2_H_3_O_2_) [M − H−C_16_H_31_]^−^ were also recorded and consistent with previous reports ^29,30^.

### Phospholipids

Phospholipids are a naturally occurring class of distinct anionic substances that are abundant in mung beans and account for 32.26% of the grains’ lipid components^31^.

Mass spectra examination allowed the characterisation of several phospholipid species mainly phosphatidylglycerols (PGs), lyso-phosphoethanolamines (lysoPEs), and lyso-phosphocholines (lysoPCs) with corresponding peaks **45**,** 48**,** 49**,** 50**,** 53**,** 60**,** 67**,** 69**,** 70** and **71** (Table 1).

Peak **45** presenting a [M + H]^+^ ion at m/z 518.2 was characterized as lysoPC 18:3. During its mass analysis, two intense product ions at m/z 239 and 257 acquired from the losses of linolenoyl acyl substituent (18:3) as free fatty acid and as ketene, respectively. The characteristic fragments at m/z 279, 235, and 59 further verified linolenic acid as the bound fatty acid.

Similarly, lysoPE at peak **48** revealed a dominating [M + H]^+^ ion at m/z 454.6. Its MS/MS profile revealed two signals at m/z 257 and 213 affirming the occurrence of palmitic acid as the bound fatty acid. Asides, two distinctive tandem ions at m/z 197 and 215 consistent with loss of the free acid (16:0) and ketene, respectively were recorded and further confirmed by literature data^28^. Accordingly, compound **48** could be annotated **as** lysoPE 16:0.

Moreover, PG at peak **67** formed a dominating [M-H]^−^ ion at m/z 757.15 and was assigned as C16:0 /C18:2 phosphatidylglycerol further verified by the relevant published data^32^. Taking the same fragmentation route, two major MS^2^ fragments at m/z 501 and 519 matching with elimination of the free acid (16:0) and ketene, respectively. As well, the product ions m/z 478 and 496 deriving from the comparable losses of the second fatty acyl substituent (18:2) were recorded. Further, ions respective to the fragmentation patterns of palmitic acid (16:0) and linoleic acid (18:2) were also perceived after the polar glycerol head group cleavage.

Similarly, PG at peak **71** generated an intense [M-H]^−^ ion at m/z 778.4 and was assigned as C18:0 /C18:0 phosphatidylglycerol further supported by the previous data^32^. During MS^2^ analysis, two main fragments at m/z 495 and 513 consistent with elimination of the free acid (18:0) and ketene, respectively. As well, the typical losses of the fatty acyl substituent (18:0) were recorded yielding signal at m/z 472 and 490. also, the typical fragment ions of stearic acid (18:0) and linoleic acid (18:2) were perceived after the polar glycerol head group cleavage.

### Phytosterols

Four peaks (**55**,** 58**,** 62 & 66**) in the phytosterol category were registered under the current analytical conditions.

Peak **(55**) exhibited a minor parent signal [M + H]^+^ at m/z 415.2 along with an intense ion at m/z 397 coherent with demethylation from the alkyl chain. Also, an evident fragment at m/z 273 was registered upon scission between C17/C20 of the aliphatic side chain. Two signals at m/z 161 and 107 corresponding to cleavage of the C-ring (C9/C11 and C8/14) were also observed. In alignment with the above observable data and reference standard, peak (**55**) could be annotated as of *β*-sitosterol.

Lanosterol **(58)** offered a negligible molecular ion [M + H]^+^ at m/z 397.2 alongside a considerable ion at m/z 409 formed upon H_2_O loss. In tandem mass analysis, a primary product ion at m/z 298 was assigned after cleavage across C17/C20 which in turn fragmented yielding fragment ions at m/z 95, 109 and 123. In addition, the primary cleavage of the C22–C23 bond in the side chain of sterols gave rise the ion at 69 m/z.

Analogously, peak **62** afforded a parent ion [M + H]^+^ at m/z 401.5 and a base peak of m/z 383 referable to water loss. The product signals at m/z 147 and 161 arising upon scission of ring C at C9/C11 and C8/14 were noted. As well, a minor fragment of m/z 268 was detected upon methyl radical loss from the side chain of dehydrated ion. In light of the aforementioned and consistent with earlier investigations^28^ peak **62** might be tentatively characterized as campesterol.

### Multivariate statistical analysis

#### Unsupervised pattern recognition of mung bean species using dendritic-heat map model

In this part, we conducted a comparison of the chemical profiles of various three mung species, namely red, green and black. This analysis utilized UPLC-MS/MS data alongside multivariate statistical methods. As previously shown, Fig. 1A illustrates notable differences in the chemical compositions of the various Mung species. The dendritic clustering analysis presented in the heat map (Fig. 1B) classified the samples into three distinct clusters: the first comprised samples from red, the second included green samples, and the third from black Mung species. It also showcased the varied distribution of the identified compounds across the three clusters as shown by color code variation from dark blue to dark brown gradient indicating low to high quantity, respectively. It was evident that red Mung samples had the highest amount of linolenic acid, daidzein, linolenoyl glycerol, phaseollin, biochanin A, gallocatechin, valine, vigvexin A, phenylalanine, 1-palmitoyl-2-linoleoyl-GPC (16:0/18:2), retinoic acid, vignatic acid B, quercetin retinoside, ricinoleic acid, lysoPC 16:1, tyramine, quercetin, petunidin 3- O-glucoside, glucitin, lanosterol, citric acid, gluconic acid, valylglycine, among others. In contrast, green mung samples exhibited elevated levels of vitexin, eicosapentanoic acid, hexosides of apigenin, kaempferol and formononetin, eriodictyol-O-glucoside, N-acetylproline, S-methylcysteine, viniferal, genistin and caffeic acid. Additionally, the black Mung had the highest amounts of trehalose, sucrose, LPE 16:0, PG (18:2/18:1), hydroxylinoleic acid, vignatic acid A, tamarixetin-3-O-(6’’-malonyl)glucoside, methionine sulfoxide, glutamyl methionine, campestrol, glycerophosphorylcholine, lysoPC 18:1, 9-Hydroxy-10,12,15-octadecatrienoic acid, arachidonic acid, stearidonic acid, 1-palmitoyl-2-arachidonoyl-GPC (16:0/20:4), oleic acid and laminarribiose, as shown in Fig. 1B.

#### Supervised pattern recognition of mung bean species using OPLS-DA model

**Fig. 2:**
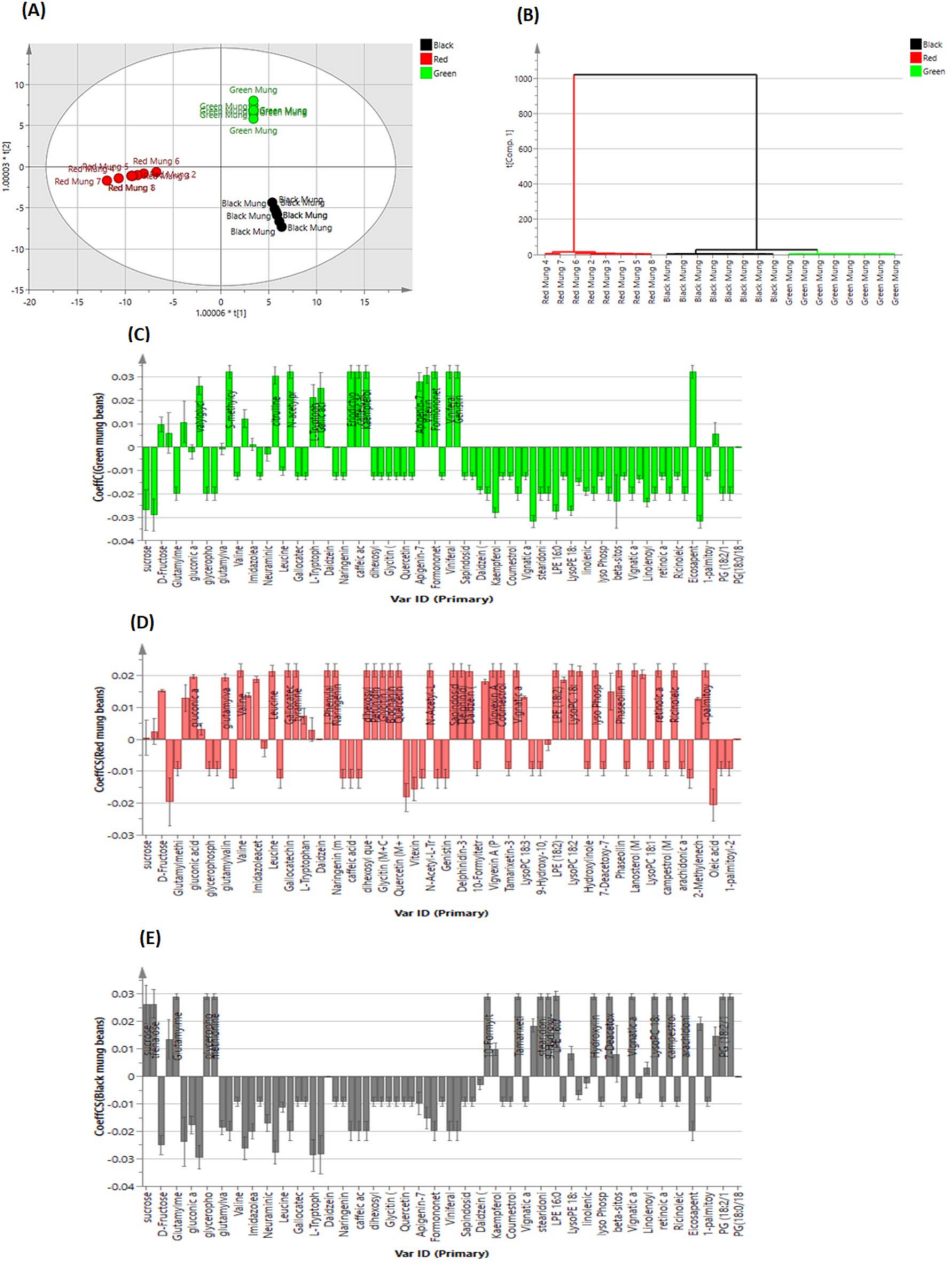
OPLS-DA score scatter plot (**A**). HCA dendrogram (**B**). Coefficient plots of the constructed OPLS-DA model for green (**C)**, red (**D**) and black (**E**) mung samples, respectively.

For the sake of inter- and intra-class differentiation of the investigated Mung bean samples, an OPLS-DA model was developed using UPLC-MS data (Fig. 2). The first and second latent variables (LVs) of OPLS-DA model accounted for 60.7% and 38.5% of the sample variability, respectively. The model showed high reliability, with a goodness of fitness (R²) of 0.995 in addition to strong predictive power with a goodness of prediction (Q²) of 0.993. The classification accuracy of the OPLS-DA model was further validated using the ROC curve shown in Fig. S2, which revealed an AUC value of 1 for all classes, confirming its excellent classification capability.

The score scatter plot (Fig. 2A) illustrated inter-class discrimination among mung bean samples along the first latent variable (LV1) where green and black mung samples clustered on the positive side indicating relative proximity in their chemical composition, while red mung samples were located on the negative side. Additionally, the second latent variable (LV2) highlighted intra-class discrimination. For instance, green mung samples were positioned on the positive side, whereas black mung samples were located on the negative LV2 side. This pattern aligned with the OPLS-DA dendrogram (Fig. 2B), which displayed two major clusters: one for green and black Mung samples, each in separate subclusters, and the other for red mung samples.

Coefficient plots were created to pinpoint specific metabolites unique to each sample, which contributed to their segregation patterns (Fig. 2C and E). The plot for the green mung samples (Fig. 2C) were characterized by S-methylcysteine, N-acetylproline, eriodictyol-O-glucoside, caffeic acid, kaempferol-3-O-glucoside, formononetin-7-O-glucoside, viniferal, genistin and eicosapentaenoic acid. In contrast, the plot for red mung samples (Fig. 2D) highlighted metabolites such as lysoPC 18:2, lanosterol, valine, gallocatechin, tyramine, phenylalanine, naringenin, dihexosylquercetin, petunidin 3- O-glucoside, glycitin, biochanin A, quercetin, quercetin rutinoside, vigvexin A, vignatic acid B, LPE 18:2, LPC 16:0, phaseollin and retinoic acid. Lastly, for the black mung samples (Fig. 2E), prominent markers included lysoPC 16:1, glutamylmethionine, S-oxide, glycerophosphorylcholine, methionine, 10-formyltetrahydrofolate, tamarixetin-3-O-(6’’-malonyl)glucoside, stearidonic acid, 9-hydroxy-10,12,15-octadecatrienoic acid, hydroxylinoleic acid, vignatic acid A, lysoPC 18:1, campestrol, arachidonic acid, PG (18:2/18:1) and 1-palmitoyl-2-arachidonoyl-GPC (16:0/20:4).

#### In vitro anti-diabetic activities of mung bean samples

Three distinct species of mung beans were evaluated for their ability to inhibit α-amylase and α-glucosidase, thereby reducing carbohydrate breakdown and glucose absorption, using in-vitro enzyme inhibition assays. As shown in (Fig. S3) the three studied mung species extracts exhibited a significant variable inhibitory effect against α-amylase, and α-glucosidase enzymes. The concentration-dependent nature of the biological effects was demonstrated by the way they increased with the extract’s concentration. In comparison to the inhibitor medication acarbose (IC_50_ = 0.431 ± 0.43 mg/ml), the red mung beans had the strongest α-glucosidase inhibitors among the studied extracts (IC_50_ = 0.178 ± 0.7 mg/ml), followed by the black mung beans (IC_50_ = 0.466 ± 0.58 mg/ml) and the green mung beans (IC_50_ = 0.6 ± 0.56 mg/ml) (Fig. S3). Regarding the α-amylase inhibitory effect, it was observed that, in comparison to the inhibitor drug acarbose (IC_50_ = 0.38 ± 0.47 mg/ml), the red mung beans had the strongest enzyme inhinition (IC_50_ = 0.248 ± 0.47 mg/ml), followed by the black mung beans (IC_50_ = 0.251 ± 0.58 mg/ml) and the green mung beans (IC_50_ = 0.297 ± 0.44 mg/ml) (Fig. S3). These findings demonstrated that the varying antidiabetic action of mung bean species could be attributed to the qualitative and quantitative differences in their phytochemical composition, particularly their phenolic content, flavonoids, and other bioactive compounds. The red mung beans showed the strongest inhibitory activity against the α-amylase and α-glucosidase enzymes, then the black, and finally the green mung beans showed the least inhibition activity.

#### Bioactivity-guided discrimination of mung bean species and identification of potential antidiabetic markers via OPLS model

**Fig. 3:**
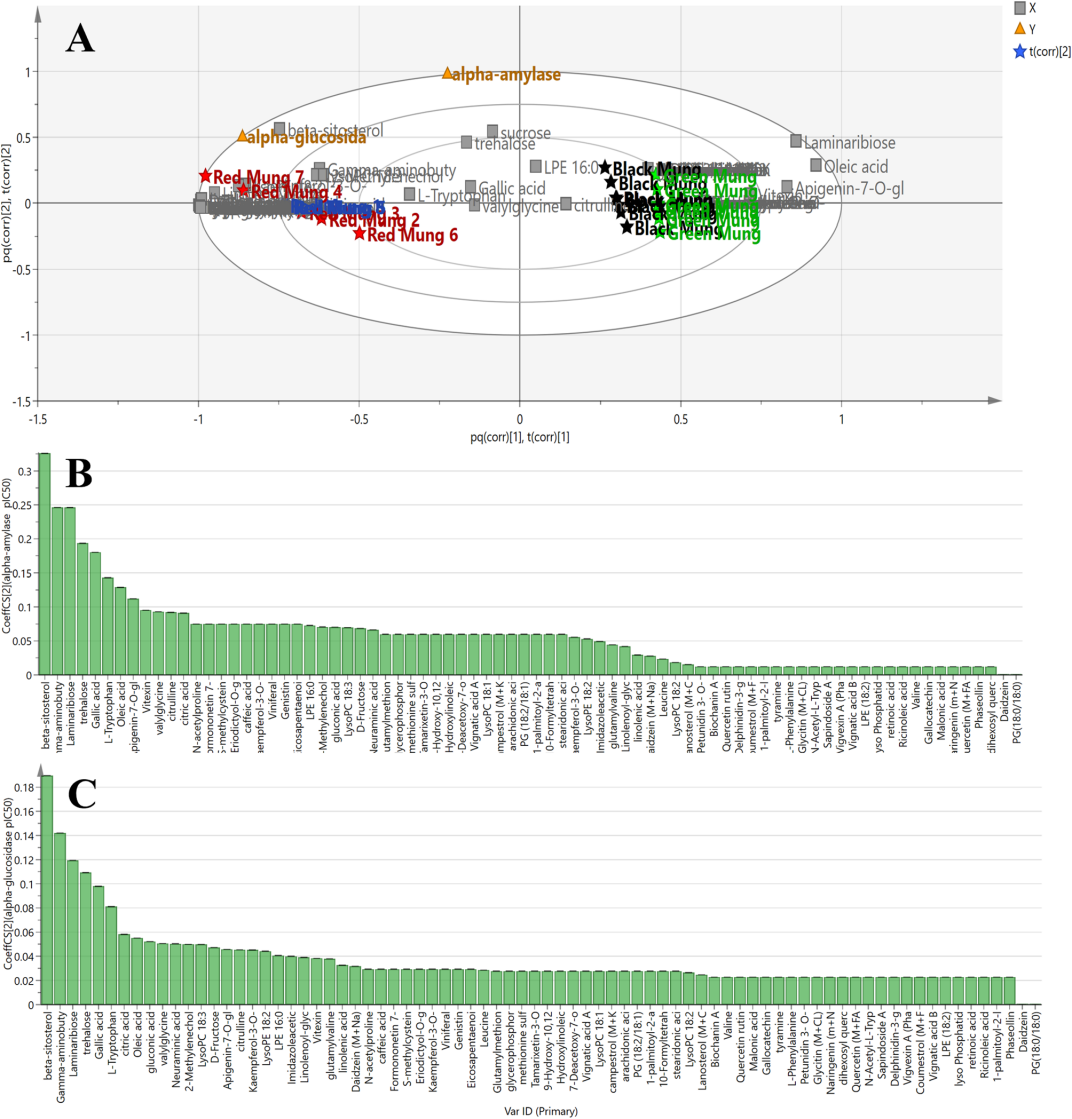
OPLS biplot chart for discrimination of mung samples according to α-amylase and α-glucosidase inhibitory activities (**A**). Coefficient plots of OPLS model revealing putative biomarkers correlated with α-amylase (**B**) and α-glucosidase (**C**) inhibitory activities.

To investigate the segregation patterns of the tested mung samples based on their anti-diabetic properties and identify the specific metabolites responsible for these effects, an OPLS model was developed. This model combined data on anti-diabetic activities (Y variables) with MS analysis data (X variables). The explained variance (R²Y) and predicted variance (Q²) for the OPLS model were found to be 0.980 and 0.985, respectively, indicating a high level of reliability. The OPLS-derived biplot (Fig. 3A) revealed that red mung samples exhibited the strongest antidiabetic activity, as they were closest to the activity data.

To pinpoint the biomarkers linked to the antidiabetic effects of the extracts, OPLS coefficient plots were generated (Fig. 3B and C). These plots highlighted key metabolites, including gamma-aminobutyric acid (GABA), gallic acid, beta-sitosterol, laminaribiose, trehalose and L-tryptophan which significantly inhibit both alpha-glucosidase and alpha-amylase activities (Fig. 3A, B and C).

The identified bioactive antidiabetic markers align with previous studies which evidenced the effective inhibitory activities of gamma-aminobutyric acid (GABA) against α-amylase and α-glucosidase enzymes^33^. Another leading investigation demonstrated that GABA administration to patients with type 2 diabetes improves insulin resistance through modulating insulin signalling pathway in skeletal muscle in diabetic patients^34^. Equally important, gallic acid has been credited with noticeable inhibitory efficacy against starch-hydrolysing enzymes, i.e. α-amylase and α-glucosidase, and serves as a promising nutraceutical product for type 2 diabetes control^35^. Another study has demonstrated that beta-sitosterol exerted noticeable inhibitory actions on α-amylase and α-glucosidase in dose dependent manner and modulated early stages of type II spontaneous diabetes in mutant mice models^36^. Essentially, previous reports have suggested that trehalose is a potential therapeutic agent to diminish blood glucose level and rectify diabetes-related complications^37^.

Our experimental findings along with OPLS analysis illuminated that these compounds stated above were possibly set as potentially efficacy compounds that function together as safe dietary inhibitors for preventing digestion of carbohydrates to control diabetes.

#### Targeted UPLC quantitation of antidiabetic biomarkers revealed from OPLS model

A UPLC method for quantification of unravelled bioactive compounds, namely; gamma-aminobutyric acid, gallic acid and beta-sitosterol; in mung samples was developed and validated in terms of specificity, linearity, limit of detection (LOD), limit of quantification (LOQ), precision, and accuracy by adhering to ICH regulatory criteria^38^. The validation parameters of the developed method are depicted in Table S1. Meanwhile, the concentration of gamma-aminobutyric acid, gallic acid and beta-sitosterol in different mung samples are represented in Table 2.

**Table 2:** Quantitation of GABA, Gallic acid and beta-sitosterol in red, black and green mung bean species (mg/g dry weight).

Sample no.		Gamma-aminobutyric acid (GABA)	Gallic acid	Beta-sitosterol
1	Red mung	3.5^ab^ ± 0.31	1.1^a^ ± 0.28	0.25 ^ac^ ± 0.03
2		3.15^a^ ± 0.33	0.99 ^a^ ± 0.15	0.22 ^ac^ ± 0.01
3		3.32^a^ ± 0.38	1.05 ^a^ ± 0.2	0.24 ^ac^ ± 0.02
4		3.85^ab^ ± 0.35	1.22 ^ab^ ± 0.23	0.28 ^ac^ ± 0.02
**5**		3.45^a^ ± 0.31	1.09 ^a^ ± 0.17	0.25 ^ac^ ± 0.04
6		2.8^a^ ± 0.29	0.88 ^a^ ± 0.11	0.29 ^ac^ ± 0.01
7		4.2^ab^ ± 0.38	1.33 ^ab^ ± 0.24	0.31 ^ac^ ± 0.03
8		3.47^a^ ± 0.31	1.09 ^a^ ± 0.2	0.25^ac^ ± 0.01
9	Black mung	1.91^ab^ ± 0.23	0.47 ^abc^ ± 0.09	0.21 ^bc^ ± 0.01
10		1.72^ab^ ± 0.2	0.42 ^abc^ ± 0.07	0.19 ^b^ ± 0.02
11		1.82^ab^ ± 0.19	0.45 ^abc^ ± 0.07	0.2 ^b^ ± 0.03
12		2.1^b^ ± 0.24	0.52 ^abc^ ± 0.08	0.24 ^bc^ ± 0.05
13		1.89^ab^ ± 0.17	0.47 ^abc^ ± 0.06	0.21 ^bc^ ± 0.04
14		1.53^abc^ ± 0.18	0.37 ^abc^ ± 0.04	0.16 ^b^ ± 0.02
15		2.29^b^ ± 0.22	0.58 ^abc^ ± 0.05	0.26 ^bc^ ± 0.03
16		1.89^ab^ ± 0.19	0.47 ^ac^ ± 0.07	0.21 ^bc^ ± 0.06
17	Green mung	3.04^bc^ ± 0.29	1.37 ^bc^ ± 0.13	0.11 ^abc^ ± 0.05
18		2.74^bc^ ± 0.26	1.23 ^bc^ ± 0.11	0.14 ^ac^ ± 0.02
19		2.89^bc^ ± 0.27	1.3 ^bc^ ± 0.12	0.103 ^abc^ ± 0.01
20		3.01^bc^ ± 0.3	1.35 ^bc^ ± 0.15	0.15 ^ac^ ± 0.06
21		2.44^c^ ± 0.23	1.09 ^c^ ± 0.1	0.12 ^abc^ ± 0.05
22		3.65^bc^ ± 0.33	1.65 ^bc^ ± 0.15	0.1 ^abc^ ± 0.08
23		3.02^bc^ ± 0.35	1.36 ^bc^ ± 0.16	0.105 ^abc^ ± 0.05
24		3.35^bc^ ± 0.38	1.51 ^bc^ ± 0.18	0.102 ^abc^ ± 0.04

*Values are average of three measurements ± SD.

Duncan’s range test was used to compare the content of the compounds among different mung species; Red mung (a), black mung (b), green mung (c).

Number with similar letter in each column are not statistically significant at 5% level.

#### NIR spectroscopic analysis of different mung samples and PLSR for prediction of quantified biomarkers

NIR has proven to be an effective tool being advantageous over other analytical techniques, including rapidity, ease of use, and price-effectiveness. It can record spectra from both solid and liquid samples with minimal pretreatment and enables the parallel analysis of multiple components^39^. However, identifying specific absorption bands linked to particular functional groups or chemical compounds can be challenging^40^.

**Fig. 4:**
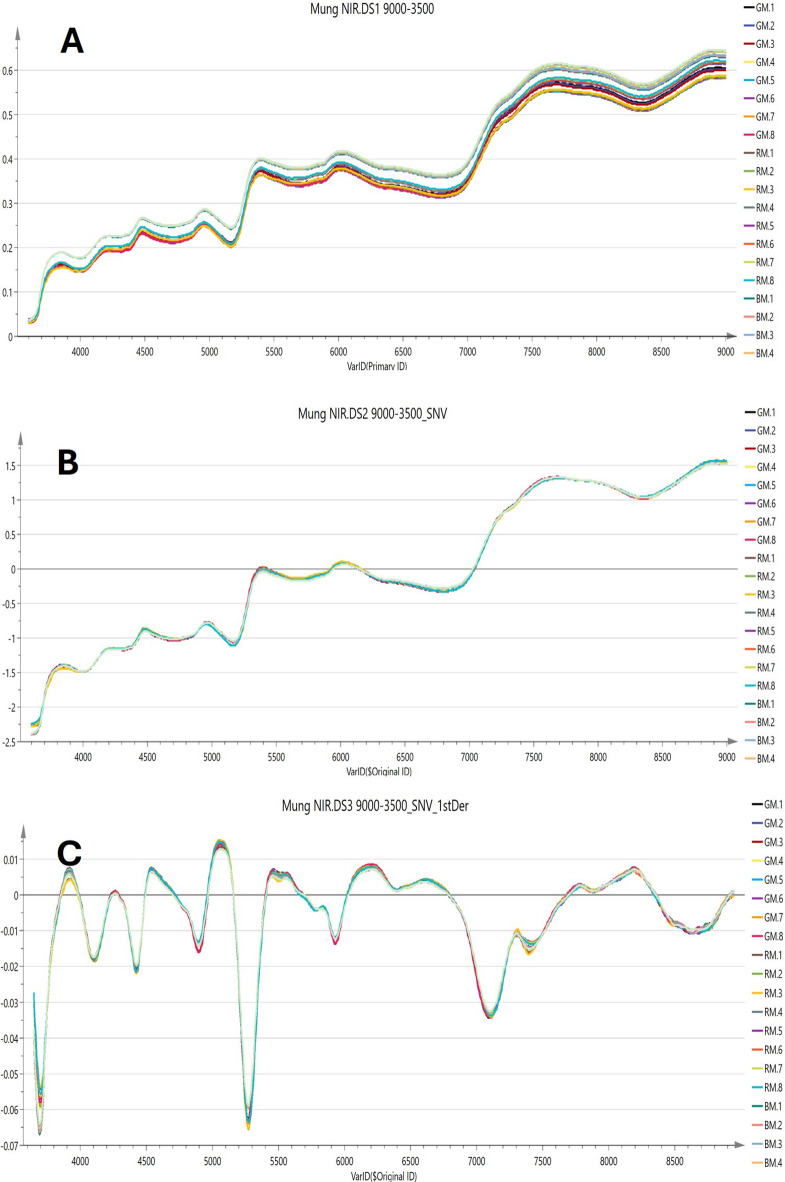
Superimposed NIR spectra of the 24 mung samples; raw spectra (**A**), after preprocessing using SNV (**B**) and 1st derivative (**C**).

**Fig. 5:**
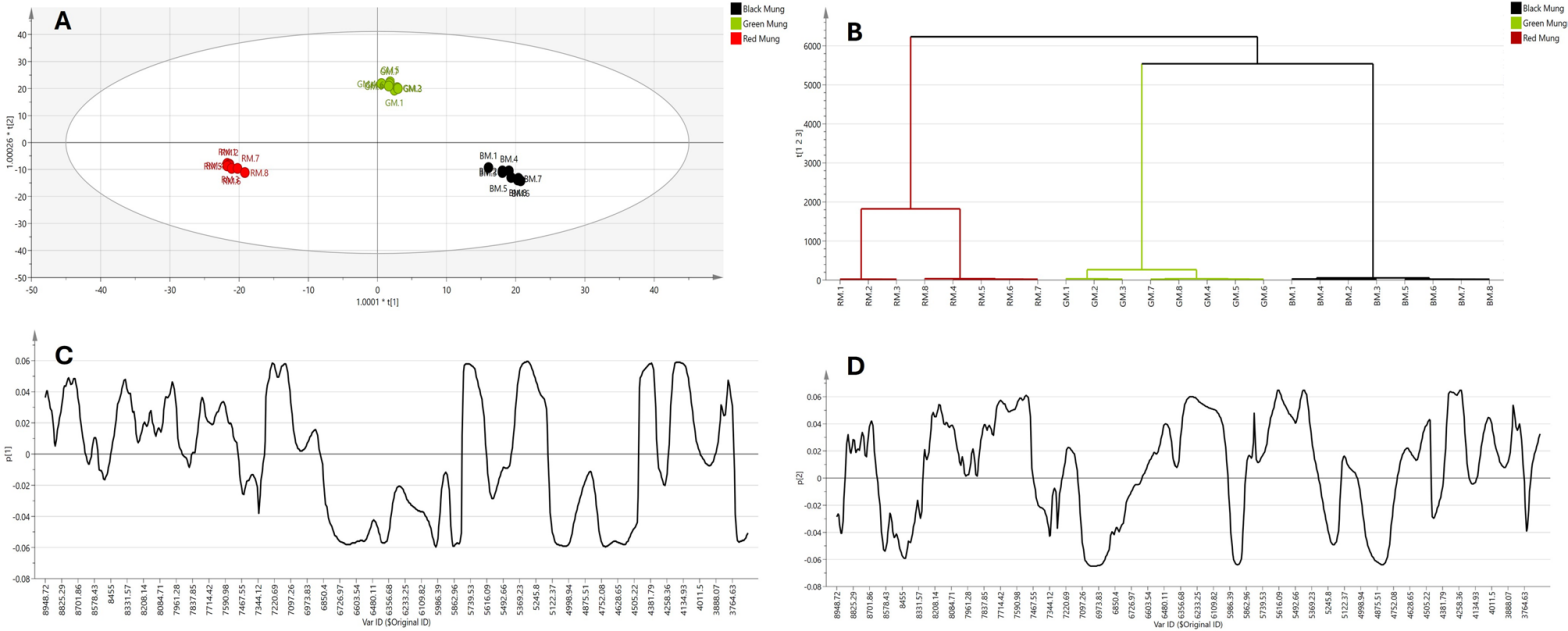
PCA score scatterplot using processed NIR data (**A**), HCA dendrogram (**B**) along with the line loadings plots of PC1 (**C**) and PC2 (**D**).

As illustrated in Fig. 4A, distinguishing between the raw NIR spectra of different mung samples is difficult because of the significant overlap of bands. However, preprocessing techniques like SNV (Fig. 4B). and first derivative treatment (Fig. 4C) of the raw spectra highlighted seven distinct absorption peaks. PCA of the processed NIR data, shown in Fig. 5A and corresponding HCA dendrogram Fig. 5B showed distinct separation between red, green and black mung samples in a manner similar to the pattern recognized from UPLC-MS data (Fig. 5A and B). The most significant wavenumbers as revealed from the loading line plots for various variables of the first and second principal components (Fig. 5C and D), respectively were identified as 4420–4366 cm^− 1^ (associated with CH stretching second overtone of –CH_2_), 5029–4990 cm^− 1^ (C = O second overtone of flavonoid nucleus)^41^, 5768–5749 cm^− 1^ (involving C-H stretching first overtone of –CH_2_, –CH_3_, –CH = CH–), 6950 cm^− 1^ (O-H first overtone of flavonoid)^42^ and 7709–7529 cm − 1 (C-H second overtone stretch vibrations models in –CH_3_)^43^.

**Fig. 6:**
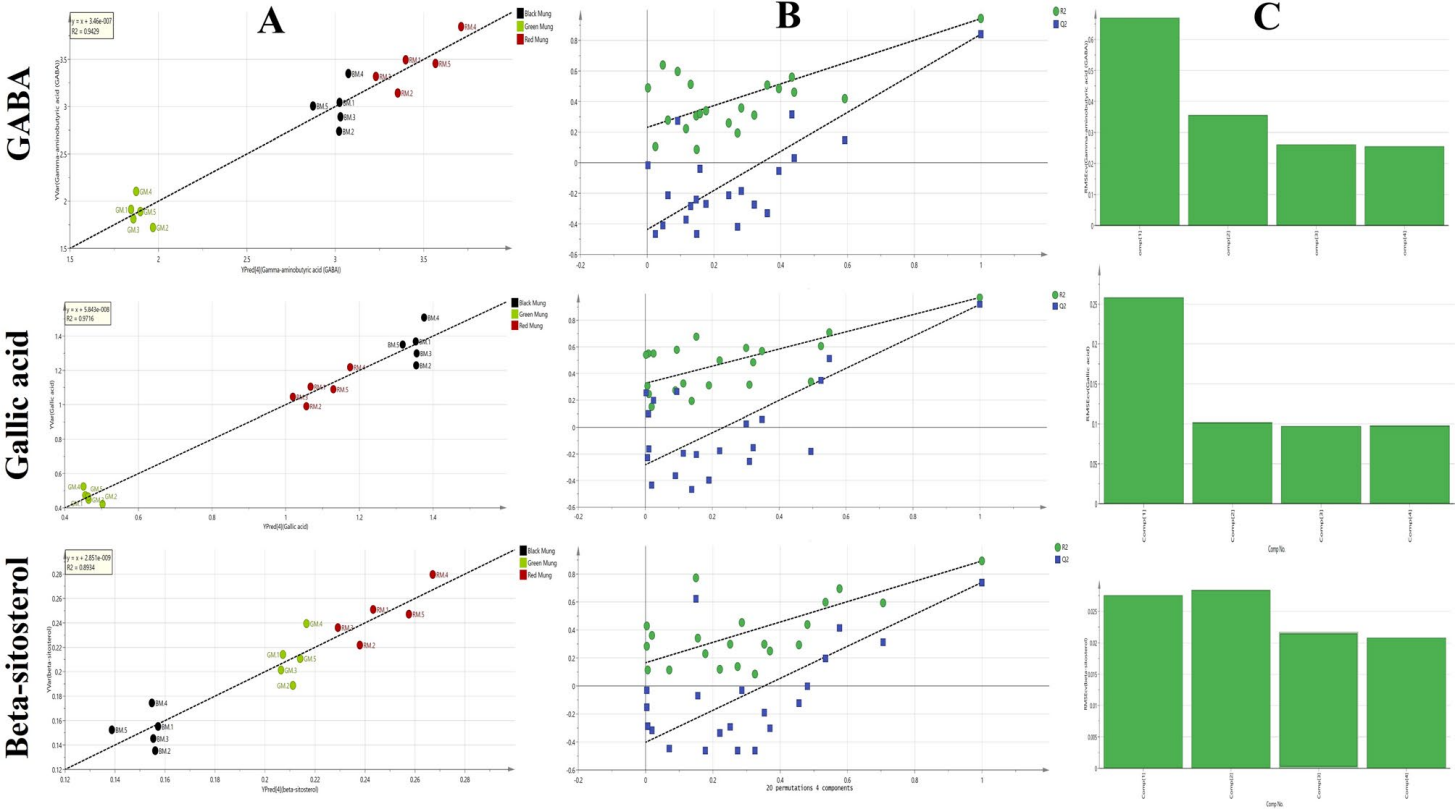
Validation of the constructed PLSR model for prediction of concentrations of GABA, gallic acid and beta-sitosterol in mung bean samples represented by; observed versus predicted plots (**A**), permutation plots (**B**) and correlation between RMSECV and optimal number of latent variables (**C**).

PLSR models were developed to predict the concentrations of identified biomarkers i.e. gamma-aminobutyric acid, gallic acid and beta-sitosterol in the samples. The samples were divided into two groups: a calibration set with 15 samples and a test set with 9 samples. First derivative of the NIR spectra from the entire range (9000–3500 cm^− 1^) served as the predictor variables (X matrix), while UPLC quantification results of the three bioactive markers were used as the dependent variables (Y matrix). NIR spectroscopy was selected as the preferred analytical method over UPLC for several reasons: it offers quicker analysis, requires fewer reagents and involves simpler procedures. The models created from the calibration samples were evaluated based on their R² values, observed versus predicted plots, and the root mean square error of calibration (RMSEC). To validate the PLS model, a LOO cross-validation method was used, where each sample is excluded in turn, allowing the model to be constructed from the remaining samples to predict the excluded one^44^. LOO cross-validation was implemented to calculate RMSECV (Table 3 and Fig. 6A) in addition to the intercept values of permutation plots (Table 3 and Fig. 6B). To assess the model’s fitting quality, we utilized RMSEE. A well-constructed PLS model is indicated by low RMSEE and RMSECV values. Moreover, it is essential to minimize the regression factors used for an effective calibration model (Table 3 and Fig. 6C). To measure the PLS model’s predictive capability, we evaluated parameters such as RMSEP^45^. Given the favourable metrics obtained in Table 3; Fig. 6, this model can reliably predict efficacy-related markers from various mung bean samples using a simple and rapid NIR run.

**Table 3:** Performance statistics of NIR-PLSR model for prediction of the three antidiabetic markers in different mung bean samples. *Values are average of three measurements ± SD.

		Gamma-aminobutyric acid	Gallic acid	Beta-sitosterol
Internal validation	R^2^	0.943	0.971	0.893
	RMSEE (mg/g)	0.200	0.077	0.016
	RMSECV (mg/g)	0.255	0.097	0.020
	Regression line equation	y = x + 3.46 e-007	y = x + 5.843e-008	y = x + 2.851e-009
	LVs	4	4	4
External validation	R^2^	0.552	0.758	0.403
	RMSEP (mg/g)	0.556	0.204	0.046
	Regression line equation	y = 0.8909x + 0.3737	y = x + 0.017	y = 0.7393x + 0.060

## Conclusion

Mung beans are common legumes consumed worldwide because of their financial and medical advantages working as a potential functional food in promoting good health. Previous studies reported their role in treatment of many metabolic illnesses, particularly type 2 diabetic mellitus (T2DM), because of its ability to reduce blood glucose levels. Therefore, in this study 24 mung bean samples collected from different red, black and green mung species have been imposed to an integrative metabolomic chemical profiling through UPLC-QqQ-MS analysis assisted by Fingerprint analysis of mung bean samples utilising Fourier Transform-Near-Infrared spectroscopy (FT-NIR). By combining these two independent techniques with multivariate analysis, a thorough and precise discrimination, along with high-quality assessment, of mung samples collected from various species was successfully achieved. A total of 71 chromatographic peaks spanning flavonoids, phenolic acids, fatty acids sugars, amino acids, and their lipid derivatives, and phytosterols were chemically profiled. Multivariable statistical models were built to reveal discriminatory compounds between red, black and green mung bean species including eriodictyol-O-glucoside, caffeic acid, formononetin-O-glucoside and genistin serve as focal discriminators of green mung beans while lysoPC 18:2, lanosterol, gallocatechin, tyramine and retinoic acid were the determining metabolites of red ones. Successively, the differential markers enriched in black mung samples included 10-formyltetrahydrofolate, stearidonic acid, vignatic acid A and PG (18:2/18:1). These compounds after being precisely identified using UPLC-QqQ-MS have been utilised to produce X-matrix of OPLS model, whereas α-amylase and α-glucosidase inhibition were allocated by the Y-matrix. The In vitro anti-diabetic assays revealed that the red beans were the most potent inhibitory activity against both α-amylase, and α-glucosidase enzymes followed by black beans and finally the green mung beans. Furthermore, it was found that gamma-aminobutyric acid (GABA), gallic acid and beta-sitosterol were the main health-relevant biomarkers. In order to predict of these biomarkers’ concentrations, a validated PLS regression model was built using FT-NIR data as predictor variables. Finally, this method may be expanded to use as a quick and easy FT-NIR run to accurately predict these active biomarkers from any red, black or green mung bean sample.

## Materials and methods

### Chemicals and reagents

The external standards: γ-Aminobutyric acid (GABA), gallic acid and β-sitosterol were acquired from Sigma-Aldrich (St. Louis, USA). Other chemicals as methanol, formic acid and acetonitrile were of analytical grade and procured from Fisher Scientific, UK and Merck India.

### Sample preparation and extraction

Three representative mung species with different bean colours (500 g), including *Vigna radiata* (green mung beans), *Vigna mungo* (black mung beans), and *Vigna angularis* (red mung beans) were selected for this study and supplied by Agricultural Research Centre, Cairo University, Egypt during September 2022. Differently coloured mung beans were vacuum freeze-dried (Edwards Lyophilizer, ModE2MB, Brazil), ground using an electric blender, and passed through a 50-mesh sieve. Subsequently, 100 g of each powder was weighed and kept in a desiccator until NIR analysis which the remaining fractions (400 g) of each mung variety were subjected to extraction process according to previous protocols ^30,46^. In short, 800 ml of 70% ethanol was used to extract 400 g of each lyophilised mung powder twice, using an ultrasonic bath apparatus (3 L Alpha Plus, Japan) at 35 °C for 60 min. Using a rotary evaporator (BuchiRotavapor Model R-200, Flawil, Switzerland), the different extracts were vacuum-concentrated until dryness.

### Chemical characterization of differently coloured mung beans extracts through UPLC-QqQ-MS/MS analysis

#### Preparation of different mung samples and reference standards solutions for LC-MS analysis

In accordance with previous sample preparation protocols ^29,44^, 1 mg/mL from each mung extract were individually resuspended in methanol (HPLC-grade), filtered using a Millipore membrane and 10 µL of each sample were loaded onto the column. Similarly, stock standard solutions of γ-Aminobutyric acid (GABA), gallic acid and β-sitosterol were prepared. These stock solutions were then diluted to working concentrations ranging from 0.01 to 2 mg/mL, as shown in Table S1. Each concentration level of the standard solutions was injected three times into the chromatographic column using 5 µL aliquots. Calibration curves of reference standards were constructed wherein the analytical parameters of linearity, limit of detection (LOD) and limit of quantification (LOQ) were tracked and assessed building on ICH criteria for analytical validation^38^ (Table S1).

#### UPLC-MS/MS analysis and mass data processing

For detailed information regarding UPLC chromatographic parameters, Electrospray ionisation- triple quadrupole mass spectrometry (ESI-QqQ-MS/MS) conditions, and the method used for metabolite annotation, please refer to the supplementary materials.

The pre-processing of the generated chromatograms, features detection, integration and alignment was performed via MZmine 2.0 (http://mzmine.sourceforge.net/) software. Mass detection utilized the local maxima approach to determine the minimum signal intensity necessary for a signal to be considered a chromatographic component. Additional MS data processing techniques, including background subtraction and adjacent peak deconvolution, were employed to consolidate nearby peaks into single peaks. Spectral alignment was achieved using the join aligner technique with one minute tolerance window for retention time, allowing for the matching of corresponding peaks across different samples while accommodating variations in retention times and m/z values during chromatographic runs^47^.

Subsequently, metabolites assignment was conducted through spectral matching with reference standards data. As well, our self-built database, typical mass spectra (quasi-molecular ions and characteristic fragmentation profiles), pertinent literature data and some public metabolite databases mainly MassBank and the Dictionary of Natural Products database were exploited in order to attain a high degree of annotation confidence.

### Multivariate statistical analysis

Hierarchical cluster analysis (HCA) heat map was generated using Metaboanalyst 6.0 (http://www.metaboanalyst.ca/) to evaluate the relative quantities of annotated metabolites in different Mung species. Multivariate statistical analysis was carried out using SIMCA-P software (Version 14.0, Umetrics, Sweden), which was utilized to develop an orthogonal projection to latent structures-discriminant analysis (OPLS-DA) model for segregation of Mung species based on their chemical compositions in addition to an OPLS model for clustering based on antidiabetic activities. Additionally, OPLS correlation coefficient plots were generated to identify phytoconstituents that were significantly correlated with the in vitro biological activities under investigation.

### FT-NIR spectral acquisition and data preprocessing

Near-infrared spectra were collected by multi-purpose Fourier Transform Near Infrared (FT-NIR) spectrometer (Bruker Optics GmbH, Ettlingen, Germany) equipped with an integrating sphere module and an InGaAs linear photodiode array detector, covering wavenumber range of 9000–3500 cm^− 1^. Spectra were recorded using OPUS software (version 6.5, Bruker Optics Inc.) at a resolution of 16 cm^− 1^, averaging over 64 scans per spectrum. Each sample was analysed in triplicate to minimize instrumental noise, utilizing uniform glass vessel (20 × 50 mm) filled with approximately 1 g of powder. Data pre-processing involved standard normal variate (SNV) transformation then 1st derivative methods, where SNV centres and scales the data while the 1st derivative corrects baseline drifts and highlights subtle differences. These processes were executed using SIMCA 14.0 software.

### PLS-R models for predicting bioactive markers from NIR metabolome

Partial Least Squares Regression (PLS-R) serves as a vital tool for analysing multivariate data, primarily for prediction. PLS-R calibration models were developed to correlate predictor variables from the NIR metabolome with the bioactive markers found in mung samples. The X-matrix contained concentrations of bioactive markers; namely, gamma-aminobutyric acid, gallic acid, and beta-sitosterol; previously determined by UPLC in each mung sample, while the X-matrix comprised NIR spectra from 24 mung samples (9000 –3500 cm^− 1^) in accordance with a previous protocol^48^. Model accuracy was assessed using metrics such as root mean square error of estimation (RMSEE), regression coefficient (R^2^, root mean square error of prediction (RMSEP), and root mean square error of cross-validation (RMSECV). The constructed PLS model was validated through Leave-One-Out (LOO) cross-validation, systematically excluding one sample at a time to predict it using the remaining data. High R^2^ values and low RMSEE, RMSEP and RMSECV indicate a robust model, with a minimal number of regression factors preferred for effective calibration.

### In vitro anti-diabetic activity testing of the studied mung species

#### Pancreatic α-amylase inhibitory activity

With a few minor modifications, the assay was performed in compliance with a previous approved protocol^49^. The supplementary information contains the assay’s specifics.

### α-Glucosidase inhibitory activity

In this assay, slight modifications were made to the previously published method^50^. The supplementary information contains the assay’s specifics.

### Statistical analysis

The data were interpreted using the statistical software GraphPad Prism v8 (GraphPad Software, San Diego, CA, USA). The statistical analysis of the data involved one-way analysis of variance, with *P* < 0.05 being deemed statistically significant. The data are expressed as the mean ± standard deviation.

## Electronic supplementary material

Below is the link to the electronic supplementary material.

Supplementary Material 1

## Data Availability

The datasets used and/or analyzed during the current study are available from the corresponding author on reasonable request.
