# Supplementary material for: Multi-omics for unveiling potential antidiabetic markers from red, green and black mung beans using NIR-UPLC-MS/MS multiplex approach
Source: Sci Rep. 2025 Jun 1;15:19213. doi: 10.1038/s41598-025-03911-x (PMC12127463; doi:10.1038/s41598-025-03911-x)
Supplement: Supplementary file 1 — Supplementary Material 1 [file 41598_2025_3911_MOESM1_ESM.docx]

***UPLC-ESI-QqQ-MS/MS analysis***

Chemical proﬁling of differently coloured mung extracts was performed using UPLC XEVO TQD triple quadruple instrument Waters Corporation, Milford, MA01757 U.S.A. Chromatographic separation was implemented on Waters Acquity UPLC BEH C18 column (50 mm × 2.1 mm ID × 1.7 μm particle size) set up at 30^°^C. The biphasic mobile phase comprised of acidiﬁed ultrapure water (0.1% formic acid) (Phase A) and acidiﬁed acetonitrile (0.1% formic acid) (Phase B) was gradient eluted at a ﬂow rate of 0.2 ml/ min and programmed as follows: 0–3 min, 5–20% B; 3–6 min, 20–60% B; 6–12 min, 60- 60% B; 12–15 min, 60–80% B; 15–20 min, 80–100% B; 20–25 min, 100- 100% B with a post-run of 5 min to equilibrate the system. The optimized parameters for ESI interface operated in positive or negative with full scan function from m/z 50_~_1200 were as follows: capillary voltage of 3 kV, cone voltage; 35 V, the ion source temperature was 150◦C, the nebulizer (nitrogen gas) pressure was 35 psi, drying and sheath gas (N_2_) temperature was 440◦C and 350◦C, respectively. The drying and sheath gas ﬂows were applied at 900 L/h and 50 L/h, respectively. The scan time and interscan delay were set to 0.4 s and 0.1 s, respectively and the analytical run time was extended to 30 min. In MS^E^ mode, 3 collision energies of 10 eV, 20 eV and 40 eV were conducted.

Of note, 5 µl from each examined sample were pooled to generate a quality control (QC) sample for judging the stability and robustness of the analytical platform.

***In-vitro* antidiabetic activity methods**

***Alpha-glucosidase inhibitory assay***

In order to examine the impact of plant extracts on α-glucosidase activity, a minor adjustment was made to the techniques employed by Nair et al. (Nair et al., 2013). 110 µl of diluted bovine pancreatin enzyme solution (5 mg/0.5 ml in 0.1 M phosphate buffer at pH=7.4) was combined with 10 µl of plant extract (test and test blank) and DMSO (negative control) in microtiter plate wells. The microtiter plate wells were incubated for thirty minutes at 37°C. Following the incubation period, each well received 60 µl of the maltose substrate solution (1% in distilled H2O) and 100 µl of the glucose oxidase Peroxidase kit reagent (Sigma-Aldrich, St. Louis, USA). The wells were then incubated for 20 minutes at 37°C, with the addition of 100 µl of phosphate buffer at pH=7.4 for the test blank exclusively. At 490 nm, the absorbance was determined using a spectrophotometer. Furthermore, the plant extract's α-glucosidase inhibitory activity was calculated using the formula ((AC-AS)/AC) * 100) where AS is the mean absorbance of the plant extract minus the mean absorbance of the plant extract blank, and AC is the mean absorbance of the negative control. Plant extract's ability to inhibit α-glucosidase was measured using the IC50 method, where the inhibitory concentration (μg/ml) represents the 50% repression of α-glucosidase. The IC50 values were ascertained using dose-response curves, with acarbose (Sigma-Aldrich, St. Louis, USA) acting as a positive control.

***Alpha-amylase inhibitory assay***

A slightly modified version of the Tamil et al. method (Tamil et al., 2010) was used to evaluate the α-amylase inhibitory activity of the tested extracts. 110 µl of diluted α-amylase enzyme solution (5 mg/0.5 ml in 0.1 M phosphate buffer at pH= 6.9) was combined with 10 µl of plant extract (test and test blank) and DMSO (negative control) in microtiter plate wells. The microtiter plate wells were incubated for thirty minutes at 37°C. Following the incubation period, 100 µl of the glucose oxidase Peroxidase kit reagent (Sigma-Aldrich, St. Louis, USA) and 60 µl of the dextrin substrate solution (1% in distilled H2O) were added to each well. The wells were then incubated for an additional 20 minutes at 37°C (100 µl of phosphate buffer at pH=6.9 in test blank only). At 490 nm, the absorbance was measured using a spectrophotometer. In the meantime, the plant extracts' α-amylase inhibitory activity was calculated using the formula ((AC-AS)/AC)*100) where AS is the mean absorbance of the plant extract minus the mean absorbance of the plantextract blank, and AC is the mean absorbance of the negative control. Plant extract's ability to inhibit α-amylase was measured using the IC50 method, where the inhibitory concentration (μg/ml) indicates the point at which 50% of α-amylase is suppressed. The IC50 values were ascertained by using dose-response curves with acarbose (positive control).

References:

Nair, S. S., Kavrekar, V., & Mishra, A. (2013). In vitro studies on alpha amylase and alpha glucosidase inhibitory activities of selected plant extracts. *European Journal of Experimental Biology*, *3*(1), 128–132.

Tamil, I. G., Dineshkumar, B., Nandhakumar, M., Senthilkumar, M., & Mitra, A. (2010). In vitro study on α-amylase inhibitory activity of an Indian medicinal plant, Phyllanthus amarus. *Indian Journal of Pharmacology*, *42*(5), 280.

**Tables:**

**Table S1: Linearity and sensitivity parameters for standard compounds**

| **Compound** | **Linearity range (mg /ml)** | **Slope (a)** | **Intercept (b)** | **Correlation coefficient (r)** | **LOD (mg /ml)** | **LOQ (mg /ml)** |
| --- | --- | --- | --- | --- | --- | --- |
| gamma-aminobutyric acid (GABA) | 0.013-0.358 | 1.15*10^8^ | 4.05*10^5^ | 0.997 | 0.012 | 0.048 |
| Gallic acid | 0.02-0.4 | 3.07*10^7^ | 8.07*10^8^ | 0.998 | 0.009 | 0.07 |
| beta-sitosterol | 0.04-0.5 | 6.25*10^7^ | 2.51*10^6^ | 0.996 | 0.02 | 0.05 |

**For each calibration curve the equation is y= ax + b, where y is the peak area, x is the concentration of the standard (mg/g dry weight), a is the slope, b is the intercept, r the correlation coefﬁcient, LOD is the limit of detection and LOQ is the limit of quantitation.**

**Figures:**

**
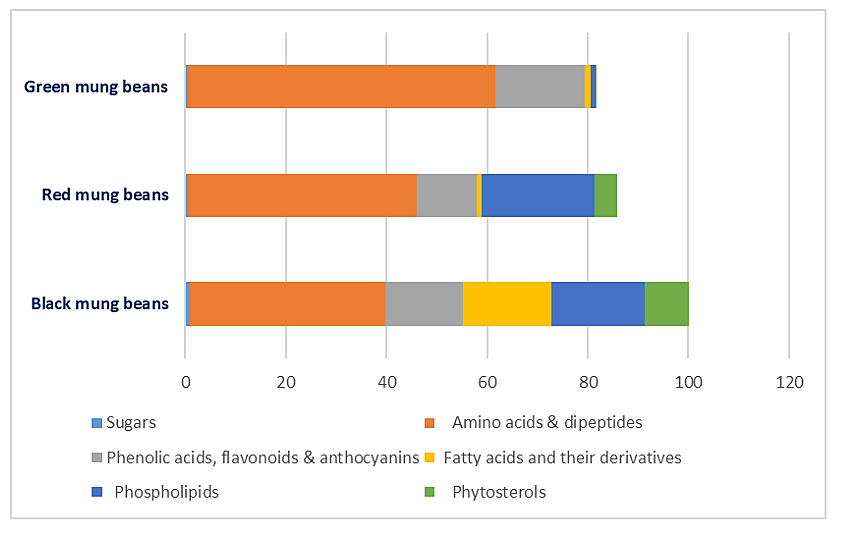
**

**Fig.S1: Stacked bar chart representing relative average abundance of main chemical classes recorded in different mung species samples.**

**
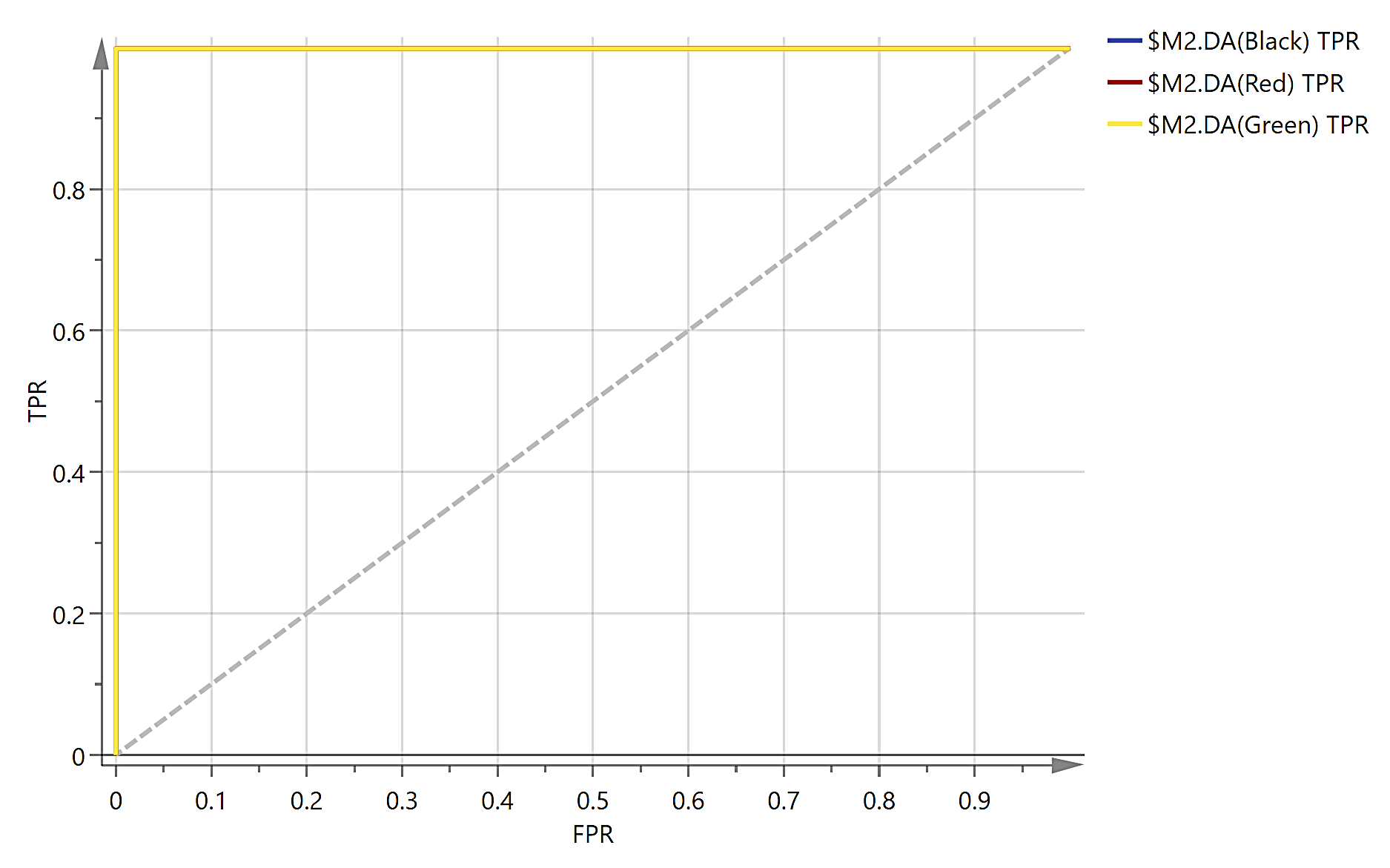
**

**Figure S2: ROC curve for OPLS-DA model validation.**


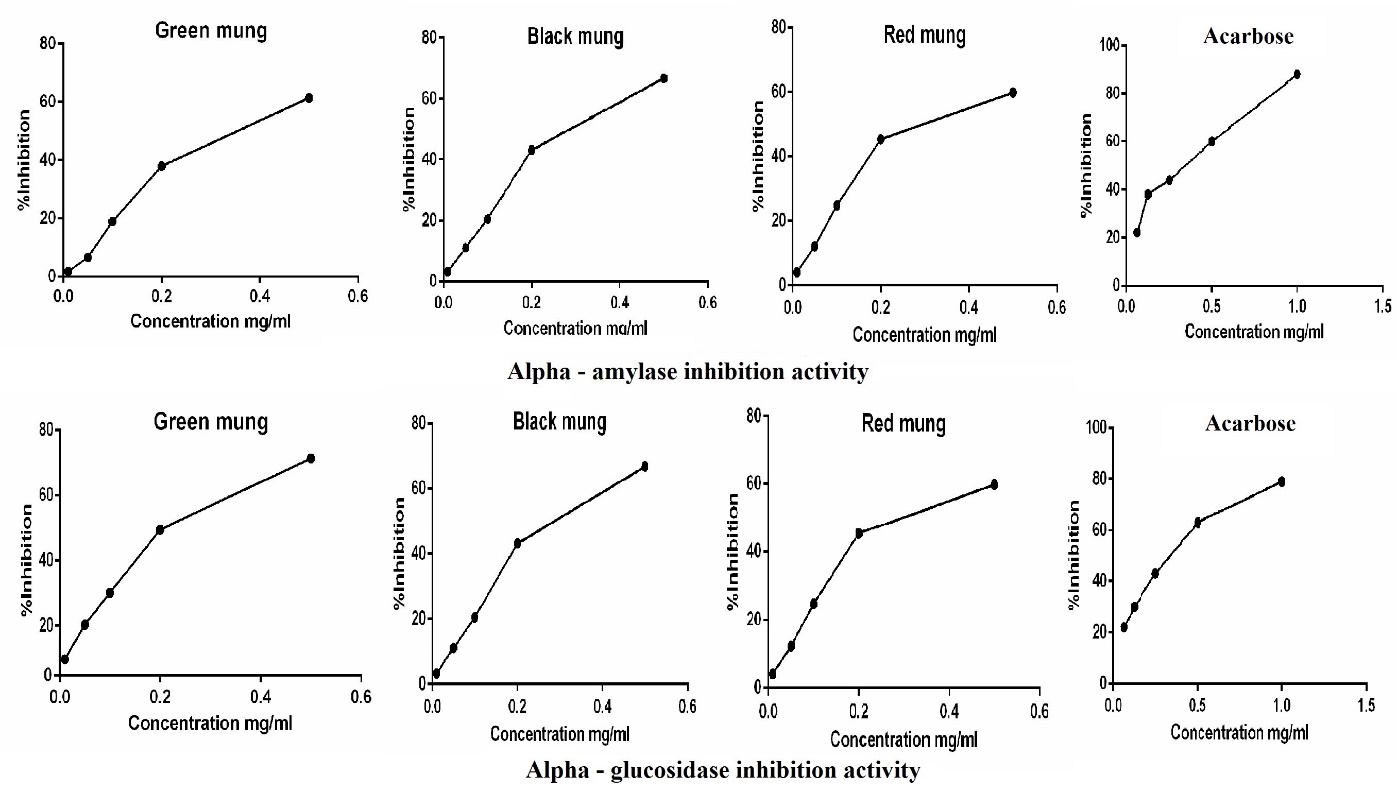


**Figure S3: Red, black, and green mung bean species' dose response curve for *in-vitro* antidiabetic efficacy against α-amylase and α-glucosidase with Acarbose as a positive control drug.**
